# Study on resting-state functional connectivity characteristics under hypnosis using functional near-infrared spectroscopy

**DOI:** 10.3389/fpsyg.2025.1567526

**Published:** 2025-06-05

**Authors:** Zhisong Zhang, Wanqiu Tan, Yuhong Ma, Min Zheng, Yuan Zhang, Jiaming Wei, Yaozu Wang, Zhimeng Li, Zhifei Li, Roger C. Ho

**Affiliations:** ^1^Faculty of Education, Huaibei Normal University, Huaibei, China; ^2^Anhui Engineering Research Center for Intelligent Computing and Application on Cognitive Behavior (ICACB), Huaibei, China; ^3^National University of Singapore (Chongqing) Research Institute, Chongqing, China; ^4^National University of Singapore (Suzhou) Research Institute, Suzhou, China; ^5^Department of Psychological Medicine, National University of Singapore, Singapore, Singapore; ^6^Institute for Health Innovation and Technology (iHealthtech), National University of Singapore, Singapore, Singapore; ^7^Division of Life Science (LIFS), Hong Kong University of Science and Technology, Kowloon, Hong Kong SAR, China

**Keywords:** functional near-infrared spectroscopy, hypnosis, frontal lobe, resting-state, functional connectivity

## Abstract

**Objective:**

Numerous studies suggest that hypnosis has significant potential in mental health and cognitive disorder treatments. However, the mechanisms by which hypnosis influences brain activity and functional network connectivity remain unclear. This study employed functional near-infrared spectroscopy (fNIRS) to investigate resting-state functional connectivity (rsFC) under hypnosis.

**Methods:**

Twenty-six healthy college students participated in the study. Resting-state oxygenated hemoglobin (HbO) data were collected from the prefrontal cortex (PFC) during both control aware and hypnotic states. Functional connectivity strengths between these states were analyzed to assess changes in brain activity associated with deep hypnosis.

**Results:**

A total of 55 paired samples *t*-tests were conducted across 11 regions of interest (ROIs), revealing statistically significant differences (*p* < 0.05) in functional connectivity strength between the control state and hypnotic state in 10 paired comparisons. Increased connectivity during hypnosis (6 pairs): LBA9-RBA10 (*t* = −2.672, *p* = 0.013), LBA6-RBA46 (*t* = −2.948, *p* = 0.007), LBA46-RBA46 (*t* = −2.516, *p* = 0.019), RBA8-RBA46 (*t* = −2.689, *p* = 0.013), RBA9-RBA46 (*t* = −2.090, *p* = 0.047), LBA10-RBA10 (*t* = −2.315, *p* = 0.029); Decreased connectivity during hypnosis (4 pairs): LBA9-LBA45 (*t* = 2.064, *p* = 0.049), LBA6-LBA45 (*t* = 3.151, *p* = 0.004), LBA8-LBA45 (*t* = 2.438, *p* = 0.022), LBA8-RBA9 (*t* = 2.085, *p* = 0.047). No significant differences were observed in connectivity strength between other ROI pairs.

**Conclusion:**

Hypnosis appears to modulate the function of the DLPFC, PFC, and related regions, enhancing specific brain network functional connectivity. This preliminary study demonstrates that resting-state functional connectivity analysis using fNIRS is a valuable approach for studying brain activity during hypnosis.

## 1 Introduction

Hypnosis is a therapeutic technique in which practitioners utilize specialized methods (e.g., relaxation and monotonous sensory stimuli) to induce a distinct altered state of consciousness distinct from sleep. Through hypnosis, individuals may access deeper unconscious processes and perceive mental content typically outside ordinary awareness. The application of hypnosis in human history spans over 250 years, tracing its origins to Franz Mesmer’s early explorations (Peter, 2024). Empirical evidence suggests that deep hypnotic states can rapidly alleviate psychological distress, enhance physical health, and improve quality of life. Hypnosis demonstrates particular efficacy in treating psychosomatic disorders and sleep-related pathologie (Chamine et al., 2018; Flammer and Alladin, 2007; Geagea et al., 2023). Notably, studies indicate heightened therapeutic responsiveness to hypnosis in children and adolescents (Rosendahl et al., 2023), potentially attributable to developmental differences in hypnotic suggestibility.

Although numerous studies have confirmed the positive effects of hypnosis in therapy and mental health, many people remain skeptical of it (Geagea et al., 2023). These misconceptions may stem from a lack of understanding or exposure to misleading information. Therefore, it is necessary to enhance public awareness and comprehension of hypnosis through further empirical research and science-based education, grounded in scientific evidence.

The advancement of neuroimaging has opened up new investigative avenues for hypnosis research. While cutting-edge techniques such as functional magnetic resonance imaging (fMRI) have been widely adopted in this domain, current neuroimaging characterizations of hypnosis demonstrate heterogeneous patterns of cerebral activation rather than a unified neural signature (Landry et al., 2017).

Hypnotic induction may correlate with increased activity in the left orbitofrontal cortex (OFC) (Aleksandrowicz et al., 2006), while hypnosis appears to enhance functional connectivity between the precuneus and the right dorsolateral prefrontal cortex (dlPFC), angular gyrus, and dorsal precuneus (Pyka et al., 2011; Wolf et al., 2022). Comparative studies on brain network connectivity during wakefulness versus hypnosis reveal significantly heightened connectivity in the dorsal attention network (DAN), salience network (SAN), and somatomotor network (SMN) during hypnotic states (Vazquez et al., 2024).

Divergent findings exist regarding default mode network (DMN) connectivity during hypnosis (McGeown et al., 2009; Sim et al., 2024), with some studies reporting reduced connectivity and others showing opposing trends (Demertzi et al., 2011).

These inconsistencies may stem from methodological factors, including fMRI’s inherent limitations (e.g., high noise levels, low motion tolerance) and variability in hypnotic protocols. Future investigations should integrate the psychosocial dimensions of hypnosis and employ multimodal neuroimaging approaches (Farahzadi and Kekecs, 2021; McGeown et al., 2009).

Functional near-infrared spectroscopy (fNIRS) is an advanced non-invasive brain imaging technology that primarily utilizes the differential absorption properties of hemoglobin in the 600–900 nm near-infrared wavelength range to measure real-time changes in cortical blood oxygen concentration, thereby indirectly reflecting brain activity. Due to its portability, high ecological validity, low cost, and tolerance to motion, fNIRS has been widely applied in brain function research (Halsband and Gerhard Wolf, 2019; Zhang et al., 2020).

Resting-state functional connectivity (rsFC) refers to the temporal synchronization of low-frequency spontaneous neural activity between different brain regions during a task-free resting state (Rainville et al., 2019; Vazquez et al., 2024). Unlike task-based analyses, rsFC does not focus on specific cognitive processes and is not constrained by external stimuli. Thus, it reveals the intrinsic functional architecture of the brain, reflecting general functional relationships between different regions (De Pascalis, 2024; Demertzi et al., 2011; Pyka et al., 2011). Resting-state functional connectivity analysis is crucial for understanding the fundamental mechanisms of brain function.

In this study, we employed fNIRS to record and observe changes in brain functional networks during hypnosis, comparing characteristic parameter differences between the control (awake) state and the hypnotic state. This approach provides scientific evidence for a deeper understanding of the intrinsic neural mechanisms underlying hypnosis.

Compared to fMRI, fNIRS offers unique advantages in hypnosis research, including higher ecological validity, long-term monitoring capability, lower cost, and easier reproducibility. In this study, we utilize fNIRS to track and analyze alterations in brain functional networks under both control and hypnotic conditions. By examining key network metrics across these states, we aim to establish a systematic framework for elucidating the intrinsic brain mechanisms of hypnosis.

## 2 Objects and methods

### 2.1 Study subjects

This study recruited 50 students (Mean age = 24.3 years, SD = 2.46 years). All participants had no history of psychiatric or neurological disorders, provided informed consent prior to the experiment, and received remuneration afterward. The study was approved by the Ethics Committee of Huaibei Normal University (Ethical Approval Number: HBSD2024-20). Of the 50 participants, 10 did not enter a hypnotic state, and 14 had poor data quality. Consequently, the final analysis included data from 26 participants.

### 2.2 Test procedure

The experimental protocol was conducted in a dedicated acoustically shielded chamber. Participants wearing functional near-infrared spectroscopy (fNIRS) devices were positioned in a semi-recumbent posture on an ergonomic recliner. The formal procedure commenced with an initial 3-min resting-state data acquisition during the controlled conscious condition. Subsequently, a certified hypnotherapist conducted a 20-min standardized hypnotic induction protocol. Following 15 min of sustained hypnosis, a 3-min silent interval was implemented to capture resting-state neural signatures under deep hypnotic state (Table 1). The session concluded with systematic dehypnotization procedures and participant debriefing.

**Table 1:** Experimental schedule table.

Experimental stages	Time parameters	Operational protocols	Data acquisition
Baseline phase	3 min	Eyes-closed rest	rsfC data collection
Hypnotic induction	10 min	Progressive relaxation technique (standardized script)	–
Hypnotic deepening	5 min	Elevator deepening technique (standardized script)	–
Resting phase	3 min	No external stimuli	rsfC data collection
Awakening	2 min	Counting awakening method	–

After the hypnosis session, the experimenter conducted verbal inquiries (such as about relaxation level, brain activity, and bodily sensations) to confirm whether the participants had entered the hypnotic state. The 10 participants who did not enter hypnosis were excluded from the data analysis.

### 2.3 fNIRS data acquisition and channel localization

**Figure 1:**
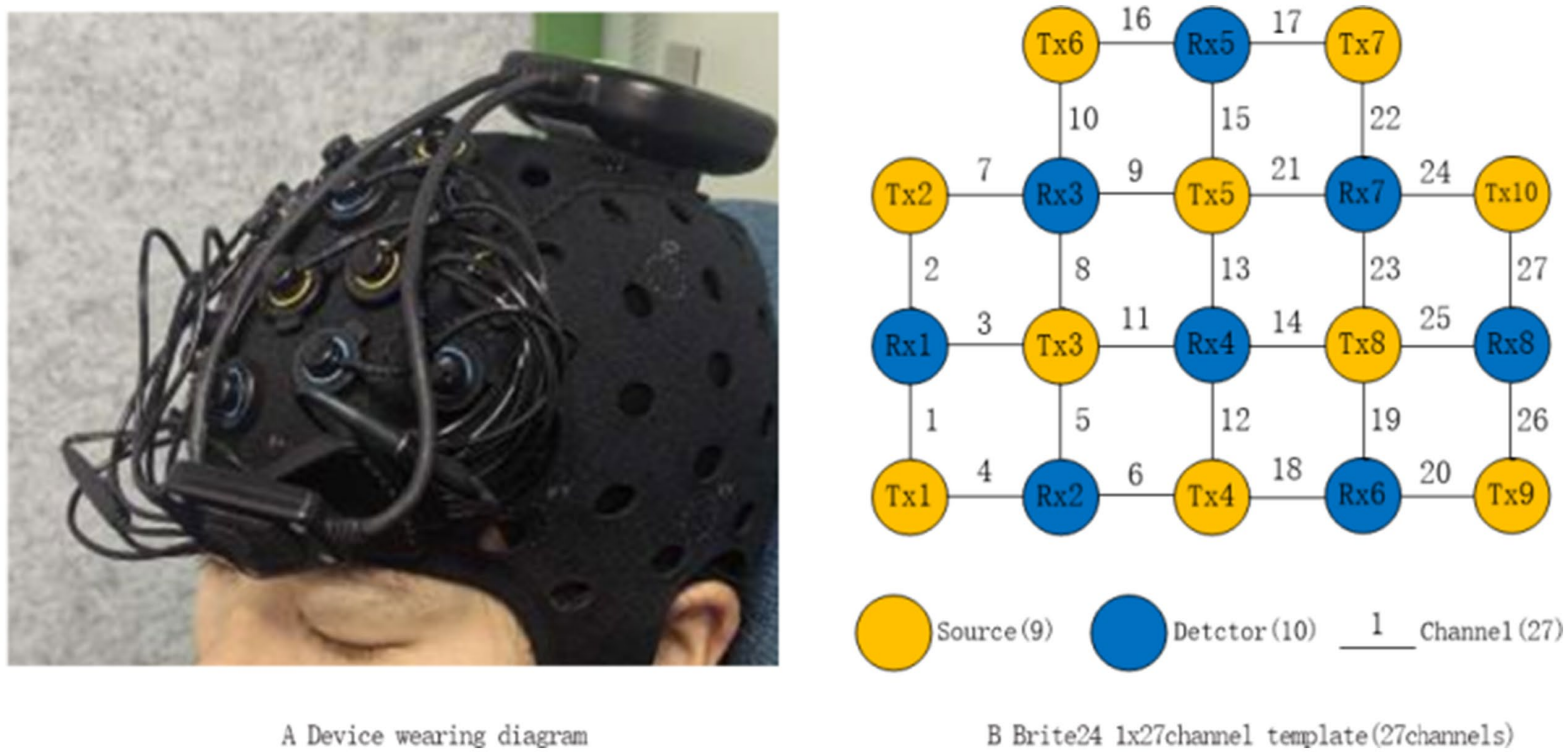
Schematic diagram of channel layout.

The fNIRS device used in this study was the Brite_24 system (Artinis Medical Systems, The Netherlands), with a sampling frequency of 25 Hz and wavelengths of 762 nm and 854 nm. The fNIRS channel layout consisted of 10 emitting electrodes (Tx1-Tx10, yellow dots) and 8 receiving electrodes (Rx1-Rx8, blue dots) for a total of 27 channels with a spacing of 3 cm (Figure 1). Data acquisition was conducted using the Oxysoft (3.4.13.1) software, and the raw light intensity signals were converted to the oxygenated hemoglobin (HbO), deoxygenated hemoglobin (HbR), and total hemoglobin (HbT) data based on the modified Beer–Lambert Law (Kocsis et al., 2006).

**Figure 2:**
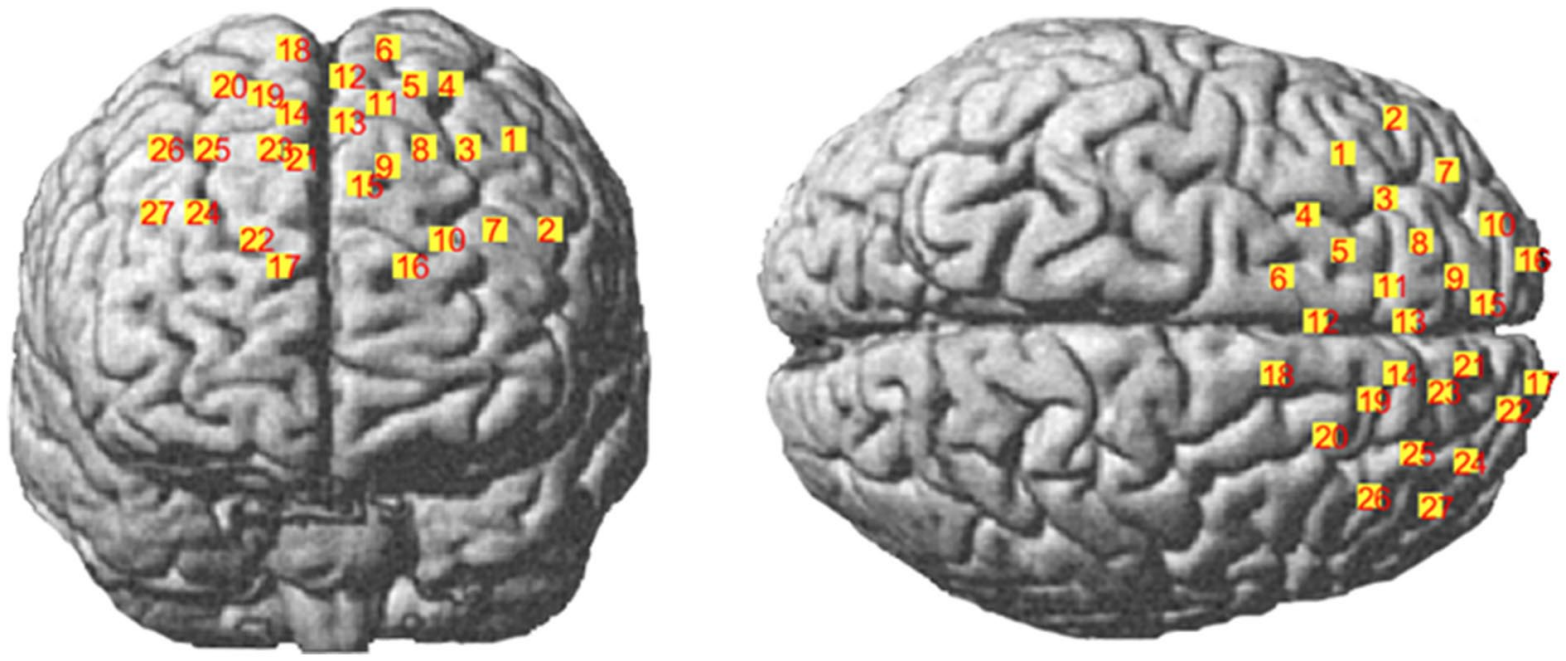
Schematic diagram of spatial coordinate localization.

The nasal root (Nz), the left anterior point (AL), the right anterior point (AR), the central point (Cz), the occipital protrusion (Iz), and the optical electrode and channel coordinates were determined by the MATLAB-based NIRS_SPM toolkit to obtain the corresponding relationship to the Brudmann partition (BrodmannArea, MRIcro) (Tables 2, 3; Figure 2) (Chul et al., 2009; Singh et al., 2005).

**Table 2:** Plot of the channel position.

Channel	Brodmann division	Coordinate MNI			Coverage probability
		*x*	*y*	*z*	
CH01	* 9-Dorsolateral prefrontal cortex	−48.74	33.56	58.39	0.89
CH02	45-Triangle District of Broca	−55.77	45.34	36.27	0.82
CH03	* 9-Dorsolateral prefrontal cortex	−39.10	44.26	58.75	0.80
CH04	* 6-Premotor cortex and the auxiliary motor cortex	−33.95	26.09	74.78	0.86
CH05	* 8-frontal eye region	−24.50	33.91	75.39	0.96
CH06	* 6-Premotor cortex and the auxiliary motor cortex	−11.52	17.87	86.65	0.98
CH07	* 46-Dorsolateral prefrontal cortex	−43.67	59.04	37.39	0.86
CH08	* 9-Dorsolateral prefrontal cortex	−25.87	54.89	58.96	0.93
CH09	* 9-Dorsolateral prefrontal cortex	−15.46	62.49	55.84	1.00
CH10	* 46-Dorsolateral prefrontal cortex	−29.48	70.49	35.29	0.56
CH11	* 8-frontal eye region	−12.56	45.23	72.50	0.94
CH12	* 6-Premotor cortex and the auxiliary motor cortex	−1.36	27.62	83.50	0.54
CH13	* 8-frontal eye region	−3.09	50.57	69.71	0.81
CH14	* 8-frontal eye region	8.70	47.74	70.93	0.87
CH15	* 9-Dorsolateral prefrontal cortex	−4.11	69.47	49.61	0.91
CH16	* 10-Prefrontal cortex	−18.06	78.53	26.91	1.00
CH17	* 10-Prefrontal cortex	11.35	79.70	27.24	1.00
CH18	* 6-Premotor cortex and the auxiliary motor cortex	9.44	17.54	86.79	1.00
CH19	* 8-frontal eye region	19.03	40.82	72.48	0.99
CH20	* 8-frontal eye region	31.44	27.71	74.22	0.92
CH21	9-found in the dorsolateral prefrontal cortex	8.64	63.79	55.81	0.98
CH22	* 10-Prefrontal cortex	21.87	73.35	34.39	0.67
CH23	* 9-Dorsolateral prefrontal cortex	18.02	58.56	58.40	0.95
CH24	* 46-Dorsolateral prefrontal cortex	35.20	63.15	39.44	0.53
CH25	* 9-Dorsolateral prefrontal cortex	34.41	50.71	55.32	0.94
CH26	* 9-Dorsolateral prefrontal cortex	44.38	38.92	56.50	0.81
CH27	* 46-Dorsolateral prefrontal cortex	46.47	52.98	38.87	0.59
	Nz	3.25	82.08	−43.36	
	Iz	−0.27	−116.63	−29.55	
	RPA	78.86	−13.09	−47.18	
	LPA	−80.87	−13.10	−46.76	
	Cz	0.65	−12.86	96.78	

* indicates region of interest (ROI, Region of Interest).

**Table 3:** Detects the brain regions and the corresponding channels.

Encephalic region	Encephalic region	Channel
*L_BA6	Premotor cortex and the auxiliary motor cortex	CH04, CH06, CH12
*R_BA6	Premotor cortex and the auxiliary motor cortex	CH18
*L_BA8	Frontal eye movement area	CH05, CH11, CH13
*R_BA8	Frontal eye movement area	CH14, CH19, CH20
*L_BA9	Dorsolateral prefrontal cortex	CH01, CH03, CH08, CH09, CH15
*R_BA9	Dorsolateral prefrontal cortex	CH21, CH23, CH25, CH26
*L_BA10	Prefrontal cortex	CH16
*R_BA10	Prefrontal cortex	CH17, CH22
*L_BA46	Dorsolateral prefrontal cortex	CH07, CH10
*R_BA46	Dorsolateral prefrontal cortex	CH24, CH27
L_BA45	Triangle District of Broca District	CHO2

CH, channel; * indicates the region of interest (ROI, Region of Interest).

### 2.4 fNIRS data analysis

#### 2.4.1 Data preprocessing

Data preprocessing was carried out using the NIRS_KIT toolkit (MATLAB R2022b) (Hou et al., 2021). The data quality was initially assessed using the Data Viewer module of NIRS_KIT, which allowed for the identification and exclusion of subject data with poor channel signal quality. A total of 14 subjects were excluded based on this criterion.

The preprocessing module of NIRS_KIT was then used to process the data as follows:

(1) First, Data Trimming: the first and last minute of the data were removed to eliminate potential edge effects.; (2) Next, Detrending: linear or nonlinear trends were estimated using polynomial regression models and then subtracted from the original hemoglobin concentration signals; (3) Motion Correction: the Temporal Derivative Distribution Repair (TDDR) method was applied to correct motion artifacts based on the time-derivative distribution; (4) Filtering: A third-order infinite impulse response (IIR) band-pass filter was applied with a frequency range set between 0.01 and 0.08 Hz to remove general noise, including heartbeat, breathing, and Merwave (Xu et al., 2023; Lu et al., 2010; Yijing Luo et al., 2024). After preprocessing, the resting-state concentration values for HbO, HbR, and HbT were obtained.

#### 2.4.2 Analysis of functional connectivity

In the Functional Connectivity module of NIRS_KIT software, each region of interest (ROI) was defined according to the groupings listed in Table 2. The average time series for each ROI was first calculated based on the relative concentration changes of HbO over time. Next, the Pearson correlation coefficient between the time series of two ROIs was computed, followed by Fisher-Z transformation. The resulting *Z* value was defined as the functional connectivity (ROI2ROI) strength between the two ROIs.

#### 2.4.3 Statistical methods

In the Group-level Statistics module of NIRS_KIT software, paired-sample *t*-test was performed on the mean value of functional connectivity strength of the two states on each brain network time series. Then, FDR (False Discovery Rate) correction method was used to correct the *p* value after multiple comparisons.

## 3 Results

After conducting paired-sample t-tests on 55 pairs across 11 ROIs, 10 pairs showed significant differences in connectivity strength between the awake and hypnotic states (*p* < 0.05). Specifically, the connectivity strength in the hypnotic state was significantly higher than in the control state for 6 pairs: LBA9-RBA10, LBA6-RBA46, LBA46-RBA46, RBA8-RBA46, RBA9-RBA46, and LBA10-RBA10. Conversely, for 4 pairs, i.e., LBA9-LBA45, LBA6-LBA45, LBA8-LBA45, and LBA8-RBA9, the connectivity strength in the hypnotic state was significantly lower than in the control state. No significant differences in connectivity strength were found for other ROI pairs (Table 4).

**Table 4:** Statistical results.

Paired channel	Paired BA	Stat	P(FDR)	Sig
CH01, CH03, CH08, CH09, CH15-CHO2	L_BA9-L_BA45	2.064	0.049	1
CH01, CH03, CH08, CH09, CH15-CH17, CH22	L_BA9-R_BA10	−2.672	0.013	1
CH04, CH06, CH12-CHO2	L_BA6-L_BA45	3.151	0.004	1
CH04, CH06, CH12-CH24, CH27	L_BA6-R_BA46	−2.948	0.007	1
CH05, CH11, CH13-CHO2	L_BA8-L_BA45	2.438	0.022	1
CH05, CH11, CH13-CH21, CH23, CH25, CH26	L_BA8-R_BA9	2.085	0.047	1
CH07, CH10-CH24, CH27	L_BA46-R_BA46	−2.516	0.019	1
CH14, CH19, CH20-CH24, CH27	R_BA8-R_BA46	−2.689	0.013	1
CH16-CH17, CH22	L_BA10-R_BA10	−2.315	0.029	1
CH21, CH23, CH25, CH26-CH24, CH27	R_BA9-R_BA46	−2.09	0.047	1

Sig value equal to 1 represents a significant difference.

## 4 Discussion

### 4.1 Hypnosis alters the functional connectivity strength in certain regions of the frontal lobe

The results of the study show that, in the hypnotic state, the connectivity strength in six pairs of regions was significantly enhanced compared to the control state. These pairs include the left dorsolateral prefrontal cortex-right prefrontal cortex (LBA9-RBA10), left premotor prefrontal cortex and auxiliary motor cortex-right dorsolateral prefrontal cortex (LBA6-RBA46), left dorsolateral prefrontal cortex-right dorsolateral prefrontal cortex (RBA46), right frontal eye region-right dorsolateral prefrontal cortex (RBA8-RBA46), right dorsolateral prefrontal cortex-right dorsolateral prefrontal cortex (RBA9-RBA46), left prefrontal cortex-right prefrontal cortex (LBA10-RBA10). These regions involve the bilateral dorsolateral prefrontal cortex (LBA9, RBA9, LBA46, RBA46), bilateral prefrontal cortex (LBA10, RBA10), left premotor cortex and auxiliary motor cortex (LBA6), and right frontal eye movement area (RBA8). This finding is consistent with the results of previous studies (Jiang et al., 2016; Vazquez et al., 2024).

The core of hypnosis lies in focused attention and relaxation. Previous studies have indicated that these brain regions, particularly the prefrontal cortex (PFC) and dorsolateral prefrontal cortex (DLPFC), are closely associated with these psychological traits. These areas play a crucial role in attention regulation, especially in inhibiting interference and selectively concentrating attention (Egner et al., 2005; Niedernhuber et al., 2024). In the hypnotic state, the high concentration of attention may be closely related to the enhanced function of these regions, which may be one of the key factors in the remarkable effect of hypnotherapy. This finding reveals the unique advantage of hypnosis in facilitating functional brain connectivity.

In addition, the study finds that connectivity is significantly reduced in four brain region pairs: the left dorsolateral prefrontal cortex-left Broca (LBA9-LBA45), left premotor cortex and auxiliary motor cortex-left Broca (LBA6-LBA 45), left frontal eye movement (LBA8-LBA45), and left frontal eye movement-right dorsolateral prefrontal cortex (LBA8-RBA9). Notably, the regions of interest (ROIs) are predominantly located in the left hemisphere. This indicates that certain areas of the left cerebral cortex, particularly the left Broca area (LBA45), responsible for speech production and motor functions, which reflects a shift in network engagement during hypnosis. These findings suggest that hypnosis may be more closely associated with the functions of the right cerebral cortex.

### 4.2 fNIRS resting-state functional connectivity analysis serves as an effective tool for investigating cerebral cortical activity during hypnotic states

Resting state functional connectivity (rsFC) analysis was designed to explore synchronized activity between brain regions during resting state. In the resting state, brain activity is spontaneous and not disturbed by external tasks, and thus, resting-state functional connectivity analysis may reveal universal properties of brain activity. Resting-state functional connectivity analysis, based on functional near-infrared spectroscopy imaging (fNIRS), is expected to be an innovative tool and method to reveal cerebral cortical activity, which deserves further exploration. This study verifies the potential of functional near-infrared spectral imaging (fNIRS) in exploring the mechanism of hypnotic brain, and provides a useful reference for subsequent studies.

### 4.3 Limitations and future work

This study primarily focused on the frontal cortex due to equipment limitations, leaving other brain regions unexamined. As a result, the findings reflect the characteristics of the selected frontal regions and do not provide a comprehensive or systematic description of whole-brain functional connectivity. Additionally, only 26 participants were included in the analysis due to signal quality issues, indicating a need for a larger sample size. Furthermore, the study exclusively involved college students, leaving functional connectivity differences across different age groups in the resting state unexplored.

Future studies could employ fNIRS devices with broader brain coverage to collect more comprehensive data, while increasing sample sizes and including participants from diverse age groups to better capture the characteristics of whole-brain functional connectivity. Additionally, enhancing signal quality will be crucial, necessitating the use of more advanced signal processing techniques and improved equipment. These advancements will enable researchers to analyze brain activity more accurately, leading to more reliable and robust conclusions.

Additionally, given that the hypnotic state may be influenced by individual psychological and physiological factors, future research should incorporate a broader range of psychological and physiological variables to examine their impact on brain functional connectivity (De Pascalis, 2023; Takarada and Nozaki, 2014). To gain a more comprehensive understanding of the neural mechanisms underlying hypnosis, it is also recommended that future studies employ multiple neuroimaging techniques for cross-validation, providing deeper and more robust scientific insights (Nia et al., 2025; Scholkmann et al., 2014).

## 5 Conclusion

Hypnosis enhances the strength of functional connectivity in certain frontal regions. Resting-state functional connectivity analysis using functional near-infrared spectroscopy (fNIRS) proves to be a valuable tool for hypnosis research. Future studies could further refine the experimental design and incorporate multimodal approaches to provide a more comprehensive investigation of neural correlates during hypnosis.

## Data Availability

The datasets presented in this study can be found in online repositories. The names of the repository/repositories and accession number(s) can be found below: https://doi.org/10.57760/sciencedb.psych.00436.
